# Ochratoxin A induces abnormal tryptophan metabolism in the intestine and liver to activate AMPK signaling pathway

**DOI:** 10.1186/s40104-023-00912-6

**Published:** 2023-09-08

**Authors:** Weiqing Ma, Yang Fu, Shanshan Zhu, Daiyang Xia, Shuangshuang Zhai, Deqin Xiao, Yongwen Zhu, Michel Dione, Lukuyu Ben, Lin Yang, Wence Wang

**Affiliations:** 1 Guangdong Provincial Key Laboratory of Animal Nutrition and Regulation, College of Animal Science, South China Agricultural University, Guangzhou, 510642 China; 2 School of Marine Sciences, Sun Yat-Sen University, and Southern Marine Science and Engineering Guangdong Laboratory (Zhuhai), Zhuhai, 519082 China; 3 College of Animal Science, YangtzeUniversity, Jingzhou, 434025 China; 4 College of Mathematics and Informatics, South China Agricultural University, Guangzhou, 510642 China; 5 Int Livestock Res Inst, 24265 Dakar, Senegal; 6 Int Livestock Res Inst, Nairobi, 00100 Kenya

**Keywords:** AMPK, Metabolomics, Ochratoxin A, Tryptophan metabolism

## Abstract

**Background:**

Ochratoxin A (OTA) is a mycotoxin widely present in raw food and feed materials and is mainly produced by *Aspergillus ochraceus* and *Penicillium verrucosum*. Our previous study showed that OTA principally induces liver inflammation by causing intestinal flora disorder, especially *Bacteroides plebeius* (*B. plebeius*) overgrowth. However, whether OTA or *B. plebeius* alteration leads to abnormal tryptophan-related metabolism in the intestine and liver is largely unknown. This study aimed to elucidate the metabolic changes in the intestine and liver induced by OTA and the tryptophan-related metabolic pathway in the liver.

**Materials and methods:**

A total of 30 healthy 1-day-old male Cherry Valley ducks were randomly divided into 2 groups. The control group was given 0.1 mol/L NaHCO_3_ solution, and the OTA group was given 235 μg/kg body weight OTA for 14 consecutive days. Tryptophan metabolites were determined by intestinal chyme metabolomics and liver tryptophan-targeted metabolomics. AMPK-related signaling pathway factors were analyzed by Western blotting and mRNA expression.

**Results:**

Metabolomic analysis of the intestinal chyme showed that OTA treatment resulted in a decrease in intestinal nicotinuric acid levels, the downstream product of tryptophan metabolism, which were significantly negatively correlated with *B. plebeius* abundance. In contrast, OTA induced a significant increase in indole-3-acetamide levels, which were positively correlated with *B. plebeius* abundance. Simultaneously, OTA decreased the levels of ATP, NAD^+^ and dipeptidase in the liver. Liver tryptophan metabolomics analysis showed that OTA inhibited the kynurenine metabolic pathway and reduced the levels of kynurenine, anthranilic acid and nicotinic acid. Moreover, OTA increased the phosphorylation of AMPK protein and decreased the phosphorylation of mTOR protein.

**Conclusion:**

OTA decreased the level of nicotinuric acid in the intestinal tract, which was negatively correlated with *B. plebeius* abundance. The abnormal metabolism of tryptophan led to a deficiency of NAD^+^ and ATP in the liver, which in turn activated the AMPK signaling pathway. Our results provide new insights into the toxic mechanism of OTA, and tryptophan metabolism might be a target for prevention and treatment.

**Graphical Abstract:**

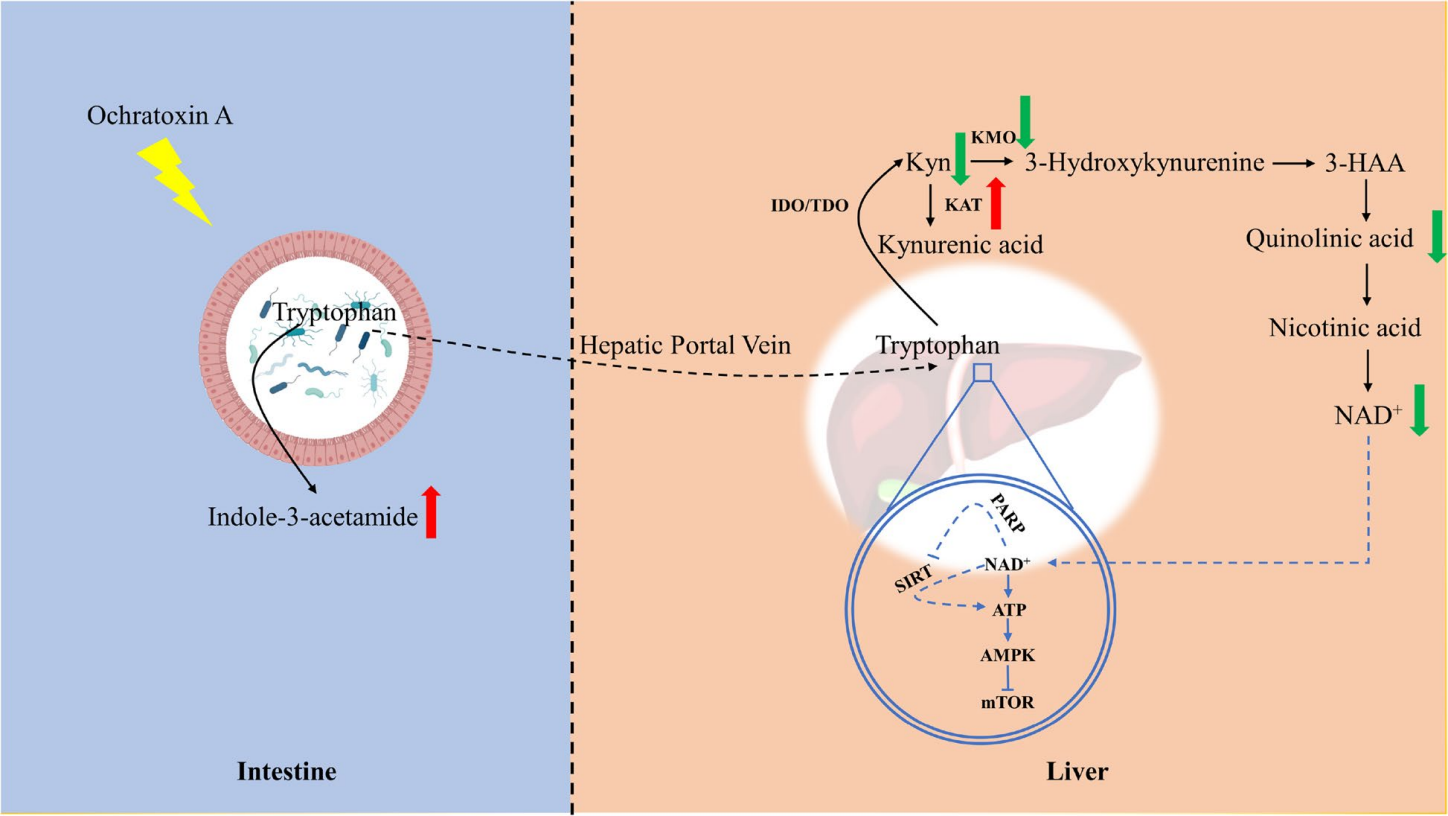

## Introduction

Ochratoxin A (OTA) is the most toxic and common form of ochratoxin and is mainly produced by *Aspergillus ochraceus* and *Penicillium verrucosum*. Its structure consists of dihydrocoumarin and *L*-β-phenylalanine linked through an amide bond [1]. As a result of its widespread presence in food and feed, OTA can enter the human body through the food consumption, causing damage to the liver, intestine, kidney and other organs, such as inhibiting liver development or inducing hepatic steatosis [2, 3]. In addition, OTA has strong teratogenicity and is defined as a class 2B carcinogen by the International Agency for Research on Cancer (IARC) [4].

The hepatorenal toxicity caused by OTA may be related to its inhibition of protein synthesis. As a result of the *L*-phenylalanine residues in its structure, OTA is a competitive inhibitor of phenylalanine tRNA synthetase, which has a greater binding ability to OTA than that of phenylalanine [5]. In addition to inhibiting cell protein synthesis, OTA also induces organ damage through autophagy and apoptosis pathways [6]. In vitro studies have shown that OTA induced an increase in calcium levels in human neutrophils, which subsequently led to depletion of ATP levels and changes in mitochondrial membrane potential [7]. As an important kinase regulating energy homeostasis, adenosine 5'-monophosphate (AMP)-activated protein kinase (AMPK) regulates metabolic activity by sensing ATP in organisms [8]. Activated AMPK promotes catabolism and reduces anabolism to decrease ATP consumption and maintain energy balance [9]. Research has shown that OTA induces phosphatase and tensin homolog deleted on chromosome ten (PTEN) activation and ATP reduction in boar sperm, thereby activating AMPK and affecting sperm motility. In addition, upregulated expression of the apoptosis markers BCL-2-associated X protein (Bax) and tumor protein 53 (p53) was found in sperm exposed to OTA [10].

The metabolic phenotypes related to gut microbiota, such as carbohydrate, amino acid, and lipid metabolism, were widely recognized by humans [11]. Tryptophan and its metabolites play an important role in regulating growth, emotion, and immunity [12]. Unlike host tryptophan metabolism, the gut microbiota can synthesize tryptophan on their own and produce metabolites that are different from those of the host. The production of the widely distributed family of tryptophanases is achieved by the gut commensal *Bacteroides* [13, 14]. In general, tryptophan is metabolized mainly through three pathways: the indole pathway, the serotonin pathway and the kynurenine pathway. Indole and its derivatives, the metabolites of tryptophan, can be used as quorum sensing signals to regulate the intestinal flora [15]. In addition, indole and its derivatives produced by the metabolism of tryptophan by intestinal microorganisms directly or indirectly activate host aromatic hydrocarbon receptors, upregulate the expression of interleukin-10 receptors, and alleviate intestinal inflammation [16]. In the liver, ninety-five percent of tryptophan is metabolized through the kynurenine pathway, and metabolites produced along this pathway, such as kynurenine, 3-hydroxyanthranilic acid (3-HAA), quinolinic acid, NAD^+^ and other active substances, participate in various metabolic processes in the body [17]. Although the metabolic pathways of tryptophan in the intestine and liver have been widely reported, the related tryptophan metabolism along the intestine-liver axis in the presence of mycotoxins has rarely been investigated.

Several studies have demonstrated that mycotoxin contamination leads to disorders of tryptophan metabolism. The activity of tryptophan 2,3-dioxygenase in the liver was reduced, and the kynurenine level was decreased in aflatoxin B1-contaminated rats [18, 19]. Nontargeted metabonomics results showed that Tibetan kefir alleviates liver injury caused by OTA via the intestinal hepatic axis via tryptophan metabolism [20].

Duck is one of the most sensitive animals to OTA, and the median lethal dose (LD50) of OTA in ducks is 0.5 mg/kg BW. The median lethal dose of OTA to pigs is 1.0 mg/kg, while the LD50 of OTA in rats and mice reaches 20–58 mg/kg BW [21–23]. Our previous study showed that OTA caused liver inflammation in ducks through the intestinal hepatic axis by increasing the abundance of the LPS-producing bacteria *B. plebeius* and therefore activating the TLR4 pathway [24, 25]. However, whether the changes in *B. plebeius* abundance induced by OTA led to abnormal tryptophan-related metabolism in the intestine and liver is largely unknown. Therefore, this experiment mainly investigated the metabolic changes in the intestinal mucosa and liver induced by OTA and tryptophan-related metabolic pathways in the liver, providing a new prevention and treatment idea for toxic damage caused by OTA.

## Materials and methods

### Animals and experimental design

A total of 30 healthy 1-day-old male Cherry Valley ducks were randomly divided into 2 groups (one was the control group and the other was the OTA group), with 15 ducks in each group, and each individual was regarded as a technical replicate. The two groups of ducks had free access to food and water, and the basal diet formulations are shown in Table 1. The prefeeding period was 7 d. From d 8, according to previous research [22, 24], the control group was given 0.1 mol/L NaHCO_3_ solution, and the OTA group was given 235 μg/kg body weight OTA (OTA was dissolved in 0.1 mol/L NaHCO_3_ solution) continuously by gavage for 14 d. Three days before the experiment, the temperature of the duck house was maintained at 32–34 °C, followed by a weekly decrease of 2–3 ºC, and was finally maintained at approximately 20 ºC. The lighting system was 24 h 10 Lux lighting. The experimental process was in line with animal ethics and was approved by the Animal Ethics Committee of South China Agricultural University (No. 20110107-1, Guangzhou, China).

**Table 1:** Ingredients composition and nutrient levels (as-fed basis)

**Ingredients**	**Content, %**	**Nutrient level**	**Content**
Cron	62.65	Crude protein, %	20.00
Soybean meal	31.625	Metabolic energy, MJ/kg	12.14
Corn gluten meal	2.30	Calcium, %	0.90
Calcium hydrogen phosphate	1.23	Total phosphorus, %	0.65
Limestone	1.16	Available phosphorus, %	0.42
NaCl	0.30	Lysine, %	1.10
98.5% Lysine	0.19	Methionine + Cysteine, %	0.80
99.8% Methionine	0.22		
Sodium bicarbonate	0.15		
Vitamin premix^a^	0.025		
Mineral premix^b^	0.10		
Choline chloride	0.05		
Total	100.00		

^a^Provided per kilogram of diet: vitamin A, 9,000 IU; vitamin D_3_, 1,500 IU; vitamin E, 7.5 IU; thiamine, 0.6 mg; riboflavin, 4.8 mg; pyridoxine, 1.5 mg; vitamin B_12_, 0.009 mg; folate, 0.15 mg; nicotinic acid, 20 mg

^b^Provided per kilogram of diet: Cu (CuSO_4_·5H_2_O), 8 mg; Fe (FeSO_4_·7H_2_O), 80 mg; Zn (ZnSO_4_·7H_2_O), 90 mg; Mn (MnSO_4_·H_2_O), 70 mg; Se (NaSeO_3_), 0.3 mg; I (KI), 0.4 mg

### Sample processing and indicator assays

After the ducks were sacrificed by bloodletting from the jugular vein, the liver and cecum digesta were aliquoted into sterilized cryopreservation tubes, placed in liquid nitrogen immediately, and then transferred to a –80 °C refrigerator. We selected 6 ducks with smaller individual differences for each group to measure the relevant indicators. The index ATP levels in the liver was determined using a commercial kit (A095-2-1, Nanjing Jiancheng Bioengineering Institute, Nanjing, China), and the dipeptidase, NAD^+^, PARP1, SIRT1 and SIRT3 levels were determined using enzyme-linked immunosorbent assay kits (YJ583943, YJ580923, YJ581234, YJ581354, YJ581242, Shanghai Enzyme-linked Biotechnology Co., Ltd., Shanghai, China).

### mRNA expression of tryptophan metabolism-related enzymes

RNA extraction was performed using an RNA extraction kit (B0004DP, EZBioscience, Roseville, USA), which was reverse transcribed into cDNA and subjected to fluorescence quantitative qPCR in a total volume of 10 µL. The reaction program was 95 °C for 5 min, followed by 40 cycles of 95 °C for 10 s and 60 °C for 30 s. The sequences of the target gene and internal reference gene (*β-actin*) are shown in Table 2, and 2^−ΔΔCt^ was used to calculate the fold change of the samples.

**Table 2:** Target gene and internal reference gene primer sequences

Genes	Sequences (5' → 3')	
*IDO2*	Forward	GGCGATGGGAAGGAGGAGACC
*IDO2*	Reverse	TCAGAGGGTTGGGAAGCAGGAAG
*TDO2*	Forward	AGCCCAGCCTCAGGTTTTCA
*TDO2*	Reverse	AGGGGACTCTCAGGCTTTGG
*KMO*	Forward	CCGCTCTACACAATGGTGACCTTC
*KMO*	Reverse	AGATGCCGCCTAGTCCTGCTG
*KAT1*	Forward	CGTCCTGAACTCACCCAACAACC
*KAT1*	Reverse	TCGTAGACCAGCCACTCGTACAC
*β-actin*	Forward	TCCTGCGGCATCCACGAGA
*β-actin*	Reverse	CCGCCGATCCAGACCGAGTA

### Western blot

We selected 4 ducks with appropriate parallelism for each group to detect the expression of related proteins. The tissue samples were washed with precooled PBS, and RIPA lysis buffer was added to extract total protein. After the protein concentrations were quantified, 2 μL of mark was added to the first empty well, 20 μL of denatured protein was loaded into the other wells, transferred to a PVDF membrane, and 5% nonfat milk powder prepared in PBST was used to immerse the membrane at 4 °C overnight. The primary antibody was diluted with PBST at a ratio of 1:1,000, the membrane was incubated with the primary antibody, and the HRP-labeled secondary antibody was diluted with PBST. The diluted secondary antibody (1:5,000) was incubated with the membrane at room temperature, and then the membrane was incubated with an ECL chemiluminescence solution (AWB0005, Aibiwei Biotechnology Co., Ltd., Changsha, China). β-Actin was used as an internal reference protein and the blots were imaged using a gel imaging system (ChemiScope6100, Clinx Science Instruments Co., Ltd., Shanghai, China). Image J software (National Institutes of Health, Bethesda, MD, USA) was used to analyze the results. Primary antibodies against AMPK, p-AMPK, mTOR and p-mTOR were purchased from Cell Signaling Technology (2532S, 2535S, 2972S, 5536S, Boston, MA, USA), and β-actin and secondary antibodies were purchased from Proteintech Group, Inc. (66009-1-Ig, SA00001-1, Des Plaines, IL, USA).

### Metabolomics analysis of cecal chyme

The metabolomics process of cecal chyme metabolites is as follows. After the chyme was extracted with the solvent (acetonitrile:methanol = 1:1, v/v), the supernatant was centrifuged (13,000 × *g*, 4 °C, 15 min). A 10-µL sample was analyzed by an UPLC-Q-TOF/MS system (Waters, Milford, MA, USA) equipped with an ACQUITY Premier BEH C18 column (100 mm × 2.1 mm, 1.7 µm) at 45 °C. The mobile phases consisted of 0.1% formic acid in water (solvent A) and 0.1% formic acid in acetonitrile:isopropanol (1:1, v/v) (solvent B). The mobile phase gradient conditions are as follows: 0–2 min, 95% A:5% B to 80% A:20% B; 2–8 min, 80% A:20% B to 40% A:60% B; 8–12 min, 40% A:60% B to 100% B and maintained 2 min; 14–14.5 min, 100% B to 95% A:5% B and maintained 1 min for equilibrating the systems. The mass spectrometer was equipped with an electrospray ionization (ESI) source operating in positive mode and negative mode. The optimal conditions were set as follows: source temperature, 500 °C; curtain gas (CUR), 30 psi; both ion sources GS1 and GS2, 50 psi; ion-spray voltage floating (ISVF), −4,000 V in negative mode and 5,000 V in positive mode; declustering potential, 80 V; collision energy (CE), 20–60 V rolling for MS/MS. Data acquisition was performed in data-dependent acquisition (DDA) mode. The detection was carried out over a mass range of 50–1,000 *m/z*.

### Metabolomics analysis of liver tissue

In short, the liver metabolome was extracted by adding the tissue to the solvent (methanol:acetonitrile:pure water = 2:2:1, v/v/v) and then the supernatant was centrifuged (12,000 r/min, 4 °C, 15 min) for UHPLC‒MS/MS analysis (ExionLC and QTRAP 6500+ system, Sciex, Framingham, MA, USA). The target compounds were chromatographed on an ACQUITY UPLC HSS T3 column (100 mm × 2.1 mm, 1.8 μm; Waters) at 40 °C, and injection volume was 5 μL. Mobile phase A was 0.1% formic acid in water, and mobile phase B was 0.1% formic acid in acetonitrile. The solvent gradient changed according to the following conditions: 0–1 min, maintained 99% A:1% B; 1–5.5 min, 99% A:1% B to 70% A:30% B; 5.5–11.5 min, 70% A:30% B to 50% A:50% B; 11.5–12.6 min, 50% A:50% B to 1% A:99% B; 12.6–12.7 min, 1% A:99% B to 99% A:1% B and maintained 2.3 min. Typical ion source parameters were as follows: curtain gas = 40 psi, ion spray voltage =  ± 4,500 V, temperature = 500 °C, ion source gas 1 = 30 psi, and ion source gas 2 = 30 psi.

### Statistical analysis

The R software package ropls (Version 1.6.2) was used to perform principal component analysis (PCA) and orthogonal least partial squares discriminant analysis (OPLS-DA) and 7-cycle interactive validation was used to evaluate the stability of the model. In addition, Student's *t*-test and fold difference analysis were performed. The selection of significantly different metabolites was determined based on the variable weight value (VIP) obtained by the OPLS-DA model and the *P* value of Student's *t*-test, and the metabolites with VIP > 1 and *P* < 0.05 were considered significantly different metabolites. The correlation was calculated by Pearson correlation analysis, and the rest of the data were analyzed by independent sample T test using SPSS 22.0 (SPSS Inc., Chicago, IL, USA). *P* < 0.05 is represented by ^*^, *P* < 0.01 is represented by ^**^, *P* < 0.001 is represented by ^***^, and *P* < 0.0001 is represented by ^****^.

## Results

### OTA treatment resulted in abnormal tryptophan metabolism in the cecal chyme

**Fig. 1:**
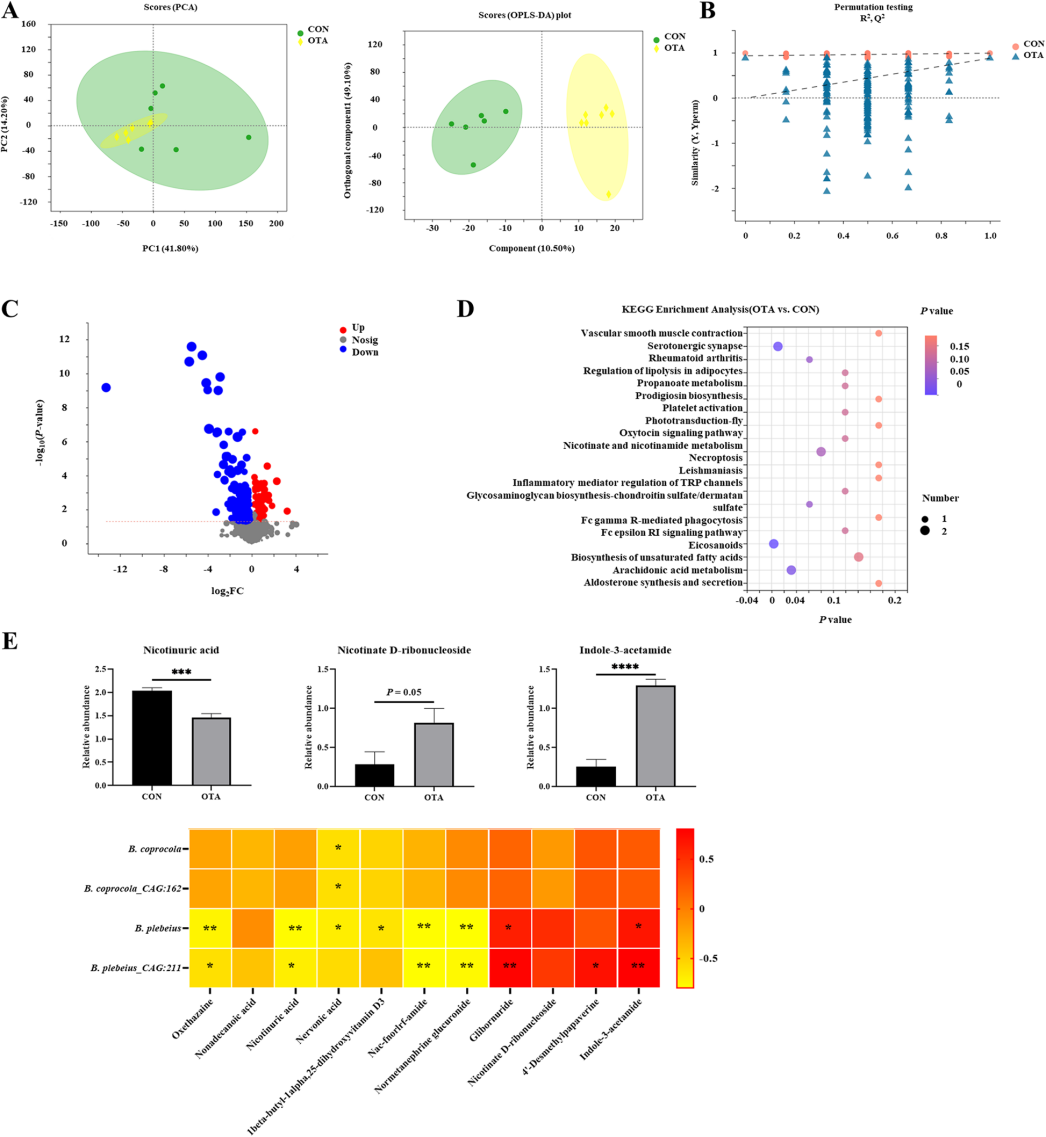
OTA caused abnormal metabolism of tryptophan in cecal chyme. **A** PCA scatter plot of all samples and OPLS-DA scatter plot of OTA vs. CON (*n* = 6). **B** OPLS-DA model permutation test for OTA vs. CON. **C** Volcano map of OTA vs. CON. **D** Differential metabolite KEGG pathway enrichment analysis.** E** Differential metabolites and their correlation with intestinal flora (*n* = 6). Values are expressed as mean ± SEM. *P* < 0.05 is represented by ^*^, *P* < 0.01 is represented by ^**^, *P* < 0.001 is represented by ^***^, and *P* < 0.0001 is represented by ^****^

Based on previous studies, we explored the relationship between the metabolome of cecal chyme and microbes under OTA treatment. As shown in Fig. 1A, the samples were all within the 95% confidence interval, and it could be seen from the OPLS-DA score map that the two groups were significantly distinguished. It could be seen from the OPLS-DA permutation test that the model had suitable robustness, and there was no overfitting phenomenon (Fig. 1B). The difference between the upregulated and downregulated metabolites between the two groups was significantly distinguished (Fig. 1C). The KEGG enrichment analysis showed that eicosanoids, serotogergic synapse signaling, arachidonic acid metabolism and nicotinate and nicotinamide metabolism were the main enriched pathways (Fig. 1D). The changes in the specific metabolites downstream of tryptophan are shown in Fig. 1E. Compared with the control group, the OTA group had significantly decreased levels of nicotinuric acid and significantly increased levels of indole-3-acetamide (IAM). The correlation analysis with intestinal bacteria showed that *B. plebeius* abundance was significantly negatively correlated with nicotinuric acid levels (*P* < 0.001) and significantly positively correlated with indole-3-acetamide levels (*P* < 0.0001).

### OTA downregulated the mRNA expression of kynurenine pathway-related enzymes in the liver

**Fig. 2:**
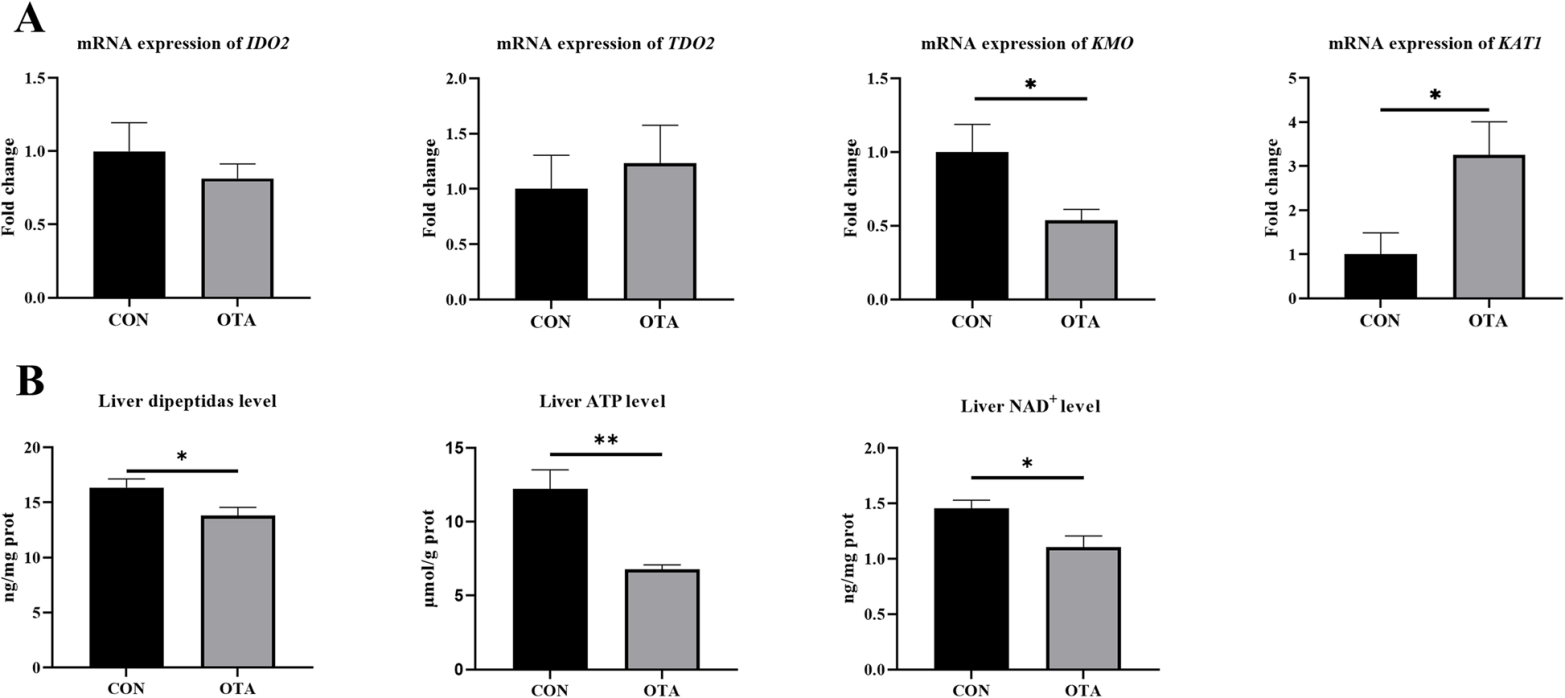
OTA decreased liver energy metabolism and enzymes expression of kynurenine pathway. **A** mRNA expression of *IDO2, TDO2, KMO* and *KAT1* (*n* = 6). **B** Dipeptidas, ATP and NAD^+^ levels in liver (*n* = 6). Values are expressed as mean ± SEM.* P* < 0.05 is represented by ^*^, *P* < 0.01 is represented by ^**^

OTA treatment significantly downregulated kynurenine-3-monooxygenase (*KMO*) mRNA expression (*P* < 0.05) and significantly upregulated kynurenic aminotransferase 1 (*KAT1*) mRNA expression (*P* < 0.05). There was no significant effect on the expression of indoleamine-2,3-dioxygenase 2 (*IDO2*) and tryptophan-2,3-dioxygenase 2 (*TDO2*) in the liver (*P* > 0.05) (Fig. 2A). Compared with the control group, the OTA group exhibited reduced levels of dipeptides (*P* < 0.05), ATP (*P* < 0.01) and NAD^+^ (*P* < 0.05) in the liver (Fig. 2B).

### OTA induced abnormal tryptophan metabolism in the liver

**Fig. 3:**
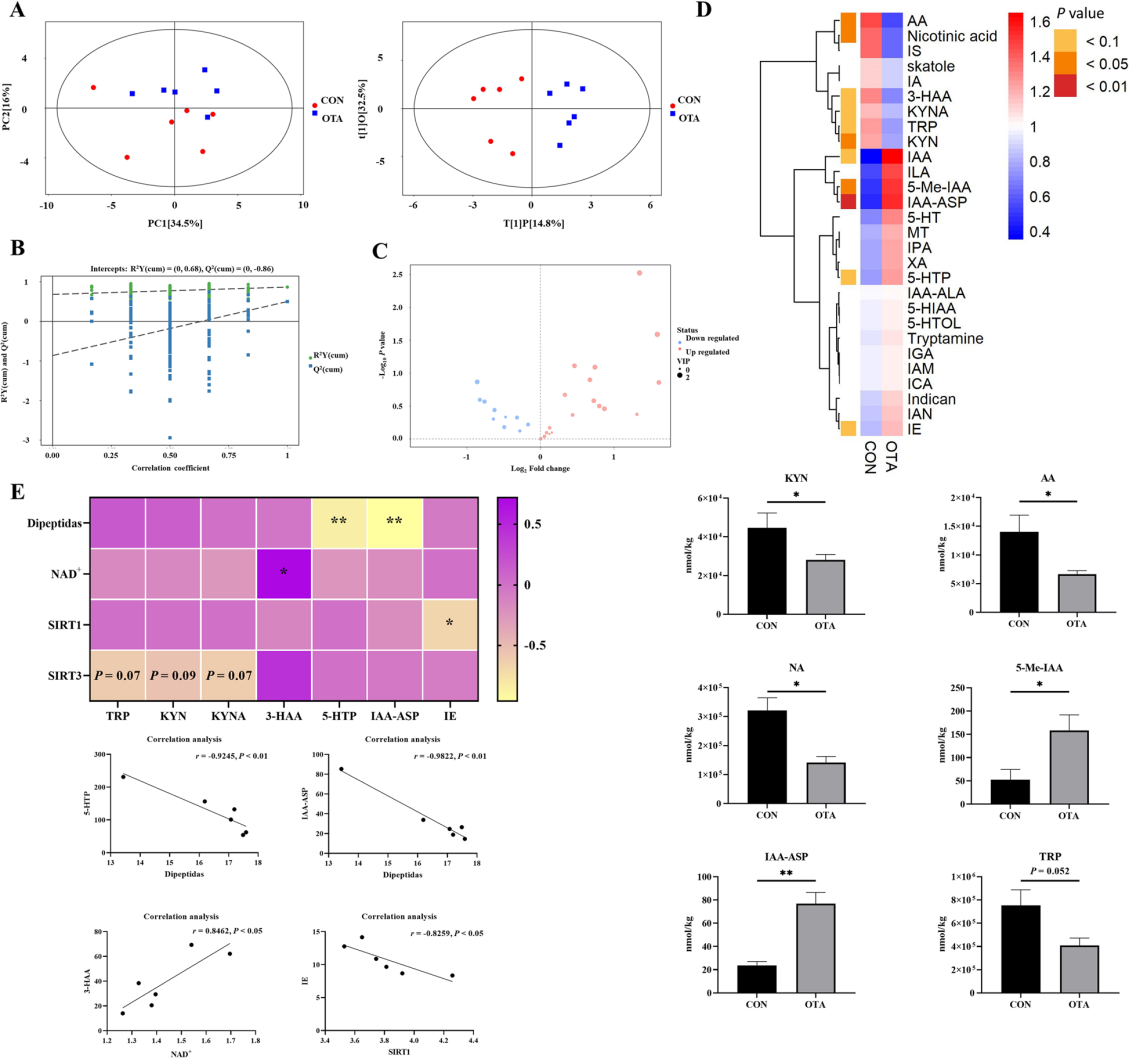
Metabonomics analysis revealed OTA reduced kynurenine metabolism in the liver. **A** PCA scatter plot of all samples and OPLS-DA scatter plot of OTA vs CON (*n* = 6). **B** OPLS-DA model permutation test for OTA vs. CON.** C** Volcano map of OTA vs. CON. **D** Differential metabolite of liver metabolomics (*n* = 6). **E** Correlation analysis of tryptophan metabolites and liver NAD^+^ related indexes. Values are expressed as mean ± SEM.* P* < 0.05 is represented by ^*^, *P* < 0.01 is represented by ^**^

To further verify the effect of OTA on hepatic tryptophan metabolism, targeted tryptophan metabolomic assays and correlation analyses were performed. The results showed that the samples were all within the 95% confidence interval, and it could be seen from the OPLS-DA score chart that the two groups were significantly different (Fig. 3A). From the OPLS-DA permutation test, we know that the model has suitable robustness (Fig. 3B). Compared with the control group, the OTA group had 18 upregulated metabolites and 10 downregulated metabolites (Fig. 3C). OTA decreased liver kynurenine (KYN), anthranilic acid (AA), and nicotinic acid (NA) levels (*P* < 0.05), increased 5-methylindole-3-acetic acid (5-Me-IAA) (*P* < 0.05) and indole-3-acetyl aspartic acid (IAA-ASP) levels (*P* < 0.01), and decreased tryptophan (TRP) levels (*P* = 0.052) (Fig. 3D). Correlation analysis showed that NAD^+^ levels were significantly positively correlated with 3-HAA levels, and dipeptide levels were significantly negatively correlated with 5-hydroxytryptophan (5-HTP) and IAA-ASP levels (Fig. 3E).

### OTA activated the AMPK signaling pathway in the liver

**Fig. 4:**
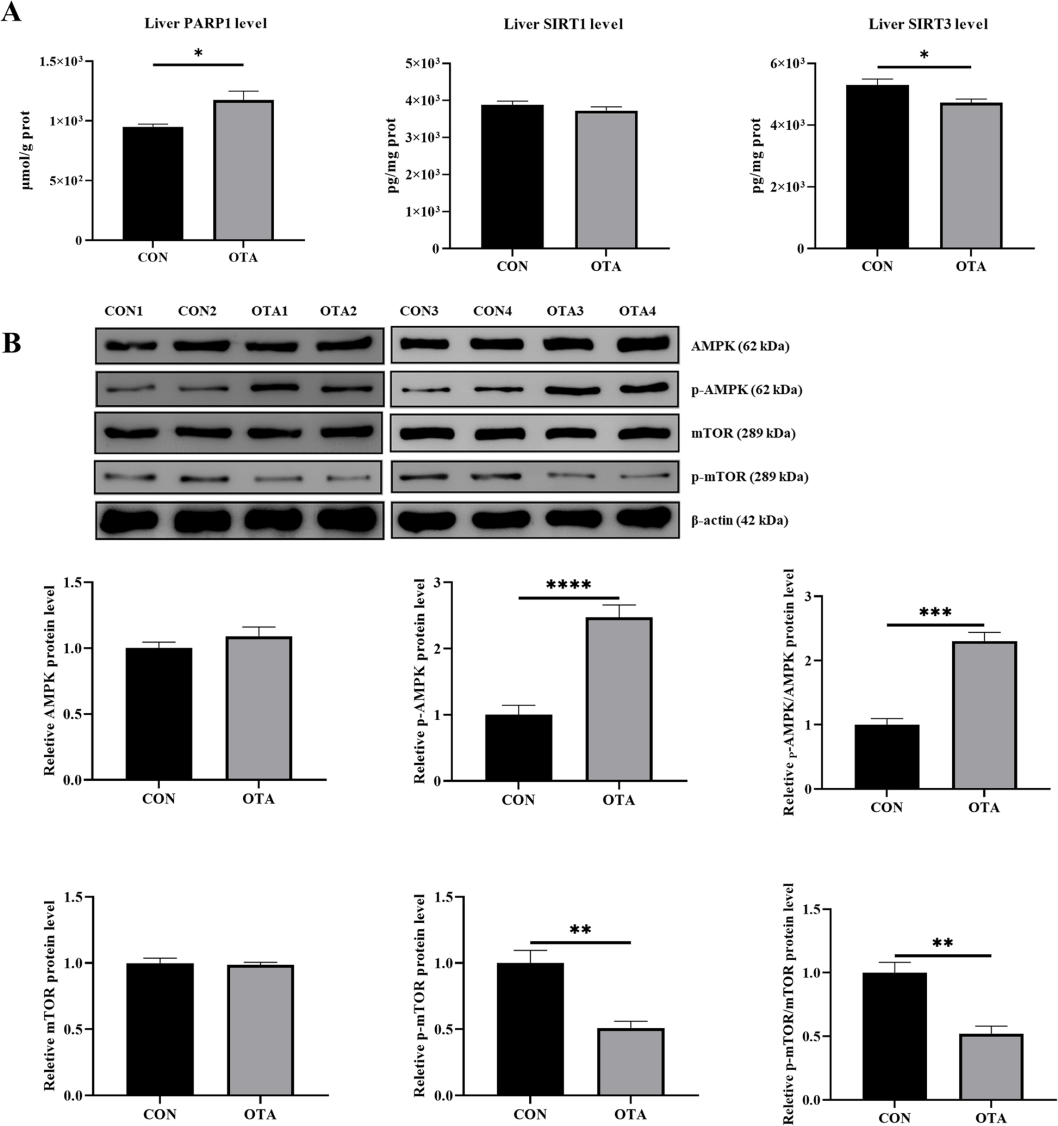
Decreased energy metabolism induced by OTA activates AMPK signaling pathway in the liver. **A** Levels of PARP1, SIRT1 and SIRT3 in the liver (*n* = 6). **B** AMPK and mTOR protein expression (*n* = 4). Values are expressed as mean ± SEM.* P* < 0.05 is represented by ^*^, *P* < 0.01 is represented by ^**^, *P* < 0.001 is represented by ^***^, and *P* < 0.0001 is represented by ^****^

OTA treatment increased liver PARP1 levels and decreased liver SIRT3 levels with no significant effect on SIRT1 levels (Fig. 4A). OTA upregulated hepatic AMPK phosphorylated protein expression and downregulated mTOR phosphorylated protein expression (Fig. 4B).

## Discussion

The mechanism of OTA-induced liver inflammation by microbial translocation through the gut-liver axis was elucidated in our previous study [24]. However, the relationship between OTA and hepatic tryptophan metabolism has not been reported in the literature. In the current study, we found that OTA-induced abnormal tryptophan metabolism in the liver is involved in gut microbiota dysbiosis. OTA decreased the level of nicotinuric acid, a product of the kynurenine metabolism pathway in intestinal chyme, which was significantly negatively correlated with *B. plebeius* abundance, and increased the level of indole-3-acetamide. Moreover, OTA inhibited the kynurenine metabolic pathway in the liver and led to deficiencies in NAD^+^ and ATP levels. The activation of the AMPK pathway and the suppression of phosphorylated mTOR protein demonstrated that OTA triggered energy deficiency in the liver.

A previous study showed that OTA disturbed the intestinal flora in the host, therefore reducing the microbial diversity and increasing the abundance of harmful bacteria [26–28]. Excessive reproduction of harmful bacteria competes for nutrients in the body, resulting in a lack of nutrients. The death and rupture of harmful Gram-negative bacteria causes LPS to seep out of the cell wall, leading to body damage [29, 30]. Notably, in addition to being an essential amino acid in the body, endogenous metabolites of tryptophan, such as kynurenine, kynurenic acid and nicotinic acid, play pivotal roles in host immunity and energy metabolism [31]. Nicotinic acid changes the polarization of macrophages from a proinflammatory state to an anti-inflammatory state, enhancing the anti-inflammatory properties of macrophages and dendritic cells in a manner dependent on G-protein-coupled receptor 109A (GPR109A) [32, 33]. Coenzymes transfer hydrogen and electrons in the redox reaction and participate in oxidative phosphorylation and DNA repair [34, 35]. As the end product of nicotinic acid and nicotinamide metabolism, nicotinuric acid can be used to reflect tryptophan-kynurenine-nicotinamide metabolism [36]. Our study found that *B. plebeius* abundance had a significant negative correlation with nicotinuric acid levels and a significant positive correlation with indole-3-acetamide levels. We speculated that the overproduction of *B. plebeius* might consume tryptophan to produce indole-3-acetamide through the indole pathway, resulting in reduced levels of nicotinuric acid, a metabolite associated with the kynurenine pathway. It was shown that the level of kynurenine in the gut and the liver is reduced, which might further inhibit kynurenine metabolism in the liver.

Ninety-five percent of tryptophan in the body is metabolized through the kynurenine pathway [12]. IDO and TDO are the rate-limiting enzymes for the metabolism of tryptophan to kynurenine. In the liver, TDO catalyzes the decomposition of tryptophan into N-formylcaninurine [37, 38]. KMO is responsible for metabolizing kynurenine to 3-hydroxykynurenine and finally to NAD^+^, which is broadly involved in energy metabolism. Kynurenine aminotransferase competes with KMO to metabolize the substrate kynurenine to kynurenic acid [17, 39]. Our study showed that OTA treatment downregulated *KMO* mRNA expression and upregulated *KAT1* mRNA expression, with no significant effect on *IDO2* and *TDO2* mRNA expression. OTA caused a significant decrease in the levels of NAD^+^ and ATP in the liver and inhibition of dipeptidase synthesis. Dipeptidase is responsible for hydrolyzing small peptides into free amino acids to complete protein hydrolysis [40]. The decrease in dipeptidase levels indicates that the level of amino acids generated from protein degradation also decreased, which forms a negative feedback process for tryptophan production [41]. The tryptophan metabolic pathway is the de novo synthesis pathway of NAD^+^. NAD^+^ achieves hydride transfer and ATP generation through mitochondrial oxidative phosphorylation [42, 43]. Abnormal metabolism causes a decrease in NAD^+^ levels, resulting in a decrease in ATP levels in the liver.

Moreover, to verify the abnormality of the tryptophan metabolic pathway, we performed metabolomic analysis of tryptophan in the liver. Interestingly, compared with the control group, OTA treatment reduced the levels of kynurenine, anthranilic acid, and nicotinic acid in the kynurenine metabolic pathway, indicating that the metabolic pathway of kynurenine was inhibited. Pearson correlation analysis showed that NAD^+^ levels were positively correlated with the levels of 3-hydroxyanthranilic acid (3-HAA). In fact, it has been shown that supplementation of tryptophan in aflatoxin-contaminated diets can alleviate the oxidative stress caused by the toxin in Japanese quail, which might be related to the upregulation of NAD^+^ [44, 45]. Organophosphorus insecticides (OPI) cause teratogenicity in chickens in the embryonic vitelline membrane by interfering with the tryptophan pathway for de novo NAD^+^ synthesis [46]. Therefore, supplementation of NAD^+^ and precursor substances in the diet may be an effective mechanism to alleviate the toxicity of OTA.

Finally, we explored the mechanism of OTA-induced liver energy metabolism damage. Poly ADP-ribose polymerase is a family of proteins with DNA repair functions, and poly-ADP-ribosylation modification is mainly completed by PARP1 [47, 48]. When DNA is damaged, poly ADP-ribose polymerase is activated, and NAD^+^ is used to synthesize poly-ADP-ribose to repair DNA [49]. Silent information regulator is an NAD^+^-dependent deacetylase that can deacetylate target proteins and regulate their activity [50]. SIRT3 migrates from the nucleus to the mitochondria and deacetylates mitochondrial proteins, therefore regulating the activity of mitochondrial proteins and ultimately affecting mitochondrial biogenesis and mitochondrial dynamics [51]. Our results showed that OTA treatment increased PARP1 levels and decreased SIRT3 levels, with no significant effect on SIRT1. This finding might indicate that OTA caused DNA damage and PARP1 activation, which consumed a large amount of NAD^+^, causing the NAD^+^ level in the liver to be insufficient. This deficiency may have inhibited SIRT3 protein expression and reduced ATP production through the impact on mitochondria [52]. Moreover, ATP reduction activated energy-regulated AMPK, and AMPK responded to its own ATP deficiency by regulating the downstream mTOR signaling pathway, reducing protein synthesis and increasing autophagy, which in turn led to an inflammatory response and weight loss in ducks during the animal experiments [29, 53–56]. Our results also showed that OTA treatment significantly upregulated the expression of phosphorylated AMPK protein and significantly downregulated phosphorylated mTOR protein expression. The weight loss in ducks might be related to the inhibition of the mTOR pathway.

## Conclusions

Our results demonstrated that the increase in intestinal *B. plebeius* abundance induced by OTA led to a decrease in the kynurenine metabolic pathway in the intestine and kynurenine metabolic pathway inhibition in the liver. The abnormal metabolism of tryptophan further led to a reduction in NAD^+^ and ATP levels in the liver, which in turn activated AMPK to inhibit the mTOR signaling pathway to counteract its own energy deficit. Our results provide new insights for the analysis of the toxic mechanism of OTA.

## Data Availability

All the datasets used and analyzed during the current study are included in the manuscript.

## Abbreviations

AA: Anthranilic acid
AMPK: Adenosine 5'-monophosphate (AMP)-activated protein kinase
ATP: Adenosine triphosphate
IAA-ASP: Indole-3-acetyl aspartic acid
IAM: Indole-3-acetamide
IDO2: Indoleamine-2,3-dioxygenase 2
KMO: Kynurenine-3-monooxygenase
KAT1: Kynurenic aminotransferase 1
KYN: Kynurenine
LD50: Median lethal dose
LPS: Lipopolysaccharides
mTOR: Mammalian target of rapamycin
NA: Nicotinic acid
NAD^+^: Nicotinamide adenine dinucleotide
OTA: Ochratoxin A
PARP1: Poly ADP-ribose polymerase 1
SIRT1: Silent information regulator 1
SIRT3: Silent information regulator 3
TDO2: Tryptophan-2,3-dioxygenase 2
TRP: Tryptophan
3-HAA: 3-Hydroxynthranilic acid
5-Me-IAA: 5-Methylindole-3-acetic acid
5-HTP: 5-Hydroxytryptophan
